# 

**Published:** 1903-03-21

**Authors:** 


### PUBLISHER'S NOTICE.

TO SUBSCRIBERS AND READERS OF "THE HOSPITAL."

### THE PRICE OF "THE HOSPITAL."

The circulation of THE HOSPITAL is now so large as to make it essential that the price shall approximate more closely to the actual cost of production than it has done in the past. It is therefore found necessary, after full consideration, to increase the price of this Journal **from 2d. to 3d. per copy**. This change in price will take effect from the first number of Volume XXXIV., which will be issued on Saturday, April 4th, 1903. The terms of subscription from that date will be as follows:—

| | Great Britain. (Post free.) | Foreign and Colonial. (Post free.) |
|---|---|---|
| For one year ... ... | 15s. 0d. | 19s. 6d. |
| For six months ... ... | 7s. 6d. | 10s. 0d. |
| For three months ... | 3s. 9d. | 5s. 0d. |

| MONTHLY PARTS. | Post free to any part of the World. |
|---|---|
| For one year ... ... ... | 15s. 6d. |
| For six months ... ... ... | 7s. 9d. |
| For three months ... ... ... | 4s. 0d. |

N.B.—Subscribers who have already paid their subscriptions will continue to receive THE HOSPITAL as issued at the old rates until such subscriptions expire.

**All new Subscriptions commencing on and after March 7, 1903, will be charged at the above Rates.**

Publishing and Editorial Offices, "The Hospital" Buildings, 28 & 29 Southampton Street, Strand, London, W.C.

### The Student of Medicine.

### Appointments.

**W. Bowie Barclay,** L.R.C.P. & S.Edin, D.P.H.Vict., appointed Medical Officer of Health for Farnborough (Hants), U.D.C. **C. Barker,** M.B.Lond., M.R.C.S., appointed District Medical Officer of the Barnet Union. **A. F. M. Berkeley,** L.R.C.P., L.R.C.S.Edin., appointed District Medical Officer of the Bath Union. **Adam G. Burrell,** M.B., M.S.Glas., appointed Public Vaccinator for the District of Rakaia, New Zealand. **Ricardo Cope,** M.R.C.S., L.R.C.P., appointed House Surgeon to the Rotherham Hospital and Dispensary. **Francis John Douglas,** M.B., appointed Public Vaccinator for Port Victor, South Australia. **Thomas Dunlop,** M.B., C.M.Edin., D.P.H.Camb., appointed Medical Officer of Health for Torquay. **J. L. Elliott,** L.S.A., appointed District Medical Officer of the Barnsley Union. **G. E. A. Evans,** M.R.C.S., L.R.C.P.Lond., appointed District Medical Officer of the Axminster Union. **Edward Alfred Graham,** M.B.Melb., appointed Visiting Surgeon to the Gaol at Deniliquin, New South Wales. **Louis S. Holmes,** L.R.C.P., L.R.C.S., L.F.P.S.G., appointed to the Honorary Medical Staff of the Launceston General Hospital, Tasmania. **C. E. M. Kelly,** M.D., M.S.Lond., appointed Certifying Factory Surgeon for the Witney District of the County of Oxford. **Benjamin Locking,** L.R.C.P.Lond., appointed Public Vaccinator for the District of Napier, New Zealand. **H. L. Maitland,** M.B., Ch.M.Syd., appointed Honorary Surgeon to the Sydney Hospital, New South Wales. **F. W. Mason,** M.R.C.S., L.R.C.P.Lond., appointed District Medical Officer of the Boston Union. **Carmelo Mifsud,** M.D., appointed Professor of Medicine at the Malta University. **U. W. M. Miles,** M.R.C.S., L.R.C.P.Lond., appointed Certifying Factory Surgeon for the Bewdley District of the County of Worcester. **James Murray,** L.R.C.P., L.R.C.S., appointed Certifying Factory Surgeon for the Allendale District of the County of Northumberland. **Jno. Orton,** M.D., Ch.B.Birmingham, M.R.C.S., L.R.C.P., D.P.H., appointed Medical Officer of Health, Toleshill Rural District, Medical Officer Toleshill Union Workhouse, and Medical Officer and Public Vaccinator Exhall and Keresley District Toleshill Union. **H. Llewellyn Porteous,** M.R.C.S., L.R.C.P.Lond., appointed District Medical Officer of the Cirencester Union. **H. A. Scholberg,** M.B.Lond, D.P.H.Camb., appointed Bacteriologist to the Glamorgan County Council and Cardiff Corporation. **W. A. R. Sharp,** M.B.Syd., appointed Medical Officer of the Coast Hospital, Sydney, New South Wales. **John C. Smith,** L.R.C.S.I., appointed Public Vaccinator for the District of Mangaweka, New Zealand. **Oliver Smithson,** M.R.C.S.Eng., L.R.C.P.Lond., appointed Medical Officer to the North Ward of the Luton Union. **A. Sprott,** M.B., C.M.Glas., appointed Certifying Factory Surgeon for the Appleby District of the County of Westmorland. **H. S. Stacy,** M.D., Ch.M.Syd., appointed Honorary Assistant Surgeon to the Sydney Hospital, New South Wales. **W. H. Thomas,** M.D.Brux., L.R.C.P.Lond., M.R.C.S.Eng., appointed District Medical Officer of the Forden Union. **Thomas Seymour Tuke,** M.B., B.Ch.Oxon., M.R.C.S. Eng., appointed Lecturer on Insanity at St. George's Hospital, W. **Arthur G. Wilkins,** M.B., Ch.B.Vict., appointed Medical Officer to the Greenside Mining Company and Medical Referee to the Prudential Assurance Company. **R. Wood,** M.D.St.And., M.R.C.S.Eng., L.R.C.P.Lond., appointed Certifying Factory Surgeon for the Barmouth District of the County of Merioneth.

The following appointments have been made at the "Dreadnought" Hospital, Greenwich:—**F. Heygate Ellis,** M.R.C.S., L.R.C.P., House Surgeon. **Frank Harvey,** M.R.C.S., L.R.C.P., Junior Resident Medical Officer. **Herman K. Burney,** B.A., Steward.

### "The Hospital" Institutional Library.

"Hospitals and Asylums of the World." 4 Vols., with a Portfolio of Plans, complete £12 12s.

"Burdett's Hospitals and Charities, 1902." 5s.

"Burdett's Official Nursing Directory, 1903." 3s. net.

"Cottage Hospitals, General, Fever, and Convalescent." 10s. 6d.

"Uniform System of Accounts for Hospitals and Public Institutions." New Edition. 4s. net.

"Hospital Expenditure: The Commissariat." 2s. 6d.

"A Legal Handbook for the Use of Hospital Authorities." 2s. 6d.

"The Cottage Hospital Case Book or Register of Patients." 10s. and 12s. 6d.

"The Furnishing and Appliances of a Cottage." 6d.

All these are published by the Scientific Press, Ltd., and may be obtained through any bookseller, or direct from the publishers, 28 and 29 Southampton Street, Strand, London, W.C.

### PUBLISHER'S NOTICE.

TO SUBSCRIBERS AND READERS OF "THE HOSPITAL."

### THE PRICE OF "THE HOSPITAL."

The circulation of The Hospital is now so large as to make it essential that the price shall approximate more closely to the actual cost of production than it has done in the past. It is therefore found necessary, after full consideration, to increase the price of this Journal **from 2d. to 3d. per copy**. This change in price will take effect from the first number of Volume XXXIV., which will be issued on Saturday, April 4th, 1903. The terms of subscription from that date will be as follows:—

| | Great Britain. (Post free.) | Foreign and Colonial. (Post free.) |
|---|---|---|
| For one year ... ... | 15s. 0d. | 19s. 6d. |
| For six months ... ... | 7s. 6d. | 10s. 0d. |
| For three months ... | 3s. 9d. | 5s. 0d. |

| Monthly Parts. | Post free to any part of the World. |
|---|---|
| For one year ... ... ... | 15s. 6d. |
| For six months ... ... ... | 7s. 9d. |
| For three months ... ... ... | 4s. 0d. |

N.B.—Subscribers who have already paid their subscriptions will continue to receive The Hospital as issued at the old rates until such subscriptions expire.

**All new Subscriptions commencing on and after March 7, 1903, will be charged at the above Rates.**

Publishing and Editorial Offices, "The Hospital" Buildings, 28 & 29 Southampton Street, Strand, London, W.C.

**"Sir Henry Burdett's monumental work on Hospitals."**

*The Times.*

A MINE OF INFORMATION ON HOSPITALS

CONSTRUCTION ORGANISATION AND MANAGEMENT

### A UNIQUE OPPORTUNITY

**FOR ALL INTERESTED IN, OR CONNECTED WITH HOSPITALS AND INSTITUTIONS.**

The result of FOURTEEN YEARS' strenuous work and an expenditure of the enormous sum of £6,500,

**for £12 12s.**

on **The Times** plan of

**TWELVE Monthly Payments of ONE GUINEA each.**

### Hospitals & Asylums of the World:

**Their Origin, History, Construction, Administration, Management, and Legislation, with Plans of the chief Medical Institutions accurately drawn to a uniform scale.**

### By SIR HENRY BURDETT, K.C.B.

In Four Volumes, with separate Portfolio (20 in. by 14 in.) containing some hundreds of Plans. Royal 8vo. cloth extra, top gilt, **£12 12s.** complete.

**Vols. I. & II. Asylums and Asylum Construction.**
**Vols. III. & IV. Hospitals and Hospital Construction.**

This valuable Encyclopædic Work contains exhaustive information on every phase of Hospital Construction, Organisation, and Management, **whilst special chapters are devoted to the History of Nursing and the Training of Nurses and Asylum Attendants.**

It is recorded that the late Mr. W. E. GLADSTONE, having read the whole of the four volumes carefully, declared it to be one of the **most interesting and remarkable works** it had ever been his good fortune to peruse.

Persons desiring to avail themselves of this Special Offer should send at once, **as only a Few Copies of the Book remain Unsold,** and this offer will be withdrawn directly these are disposed of.

PROSPECTUS AND ORDER FORM POST FREE ON APPLICATION.

THE SCIENTIFIC PRESS, LTD.,
28 & 29 Southampton Street, Strand, London, W.C.

